# Supplementary material for: Combining chemical and genetic approaches to increase drought resistance in plants
Source: Nat Commun. 2017 Oct 30;8:1183. doi: 10.1038/s41467-017-01239-3 (PMC5662759; doi:10.1038/s41467-017-01239-3)
Supplement: Supplementary file 1 — Supplementary Information [file 41467_2017_1239_MOESM1_ESM.pdf]

Supplementary Figure 1

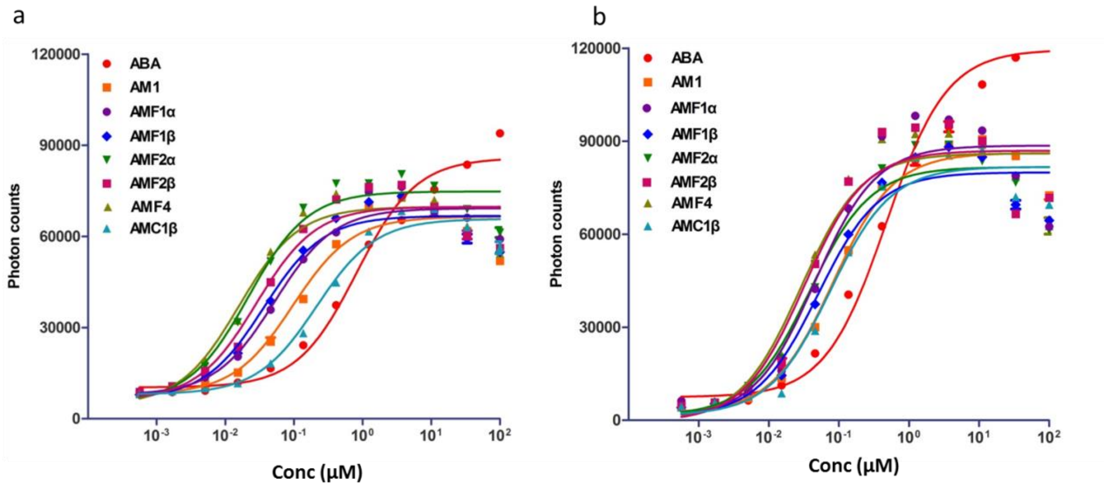

EC_{50} (nM)	ABA	AM1	AMF1α	AMF1β	AMF2α	AMF2β	AMF4	AMC1β
**GmPYL3+ AtHAB1**	820.1	96.5	52.3	36.6	21.7	28.4	15.8	212.7
**GmPYL6+ AtHAB1**	471.7	82.0	45.8	50.4	38.3	32.0	28.7	75.1

Supplementary Figure 1. Dose-response curves of the binding of AMFs and AMC1β to soybean PYLs (GmPYL) and *Arabidopsis* HAB1 (AtHAB1).

GmPYL3 (a) and GmPYL6 (b) are orthologs of AtPYL1 and AtPYL2, respectively.

Supplementary Figure 2

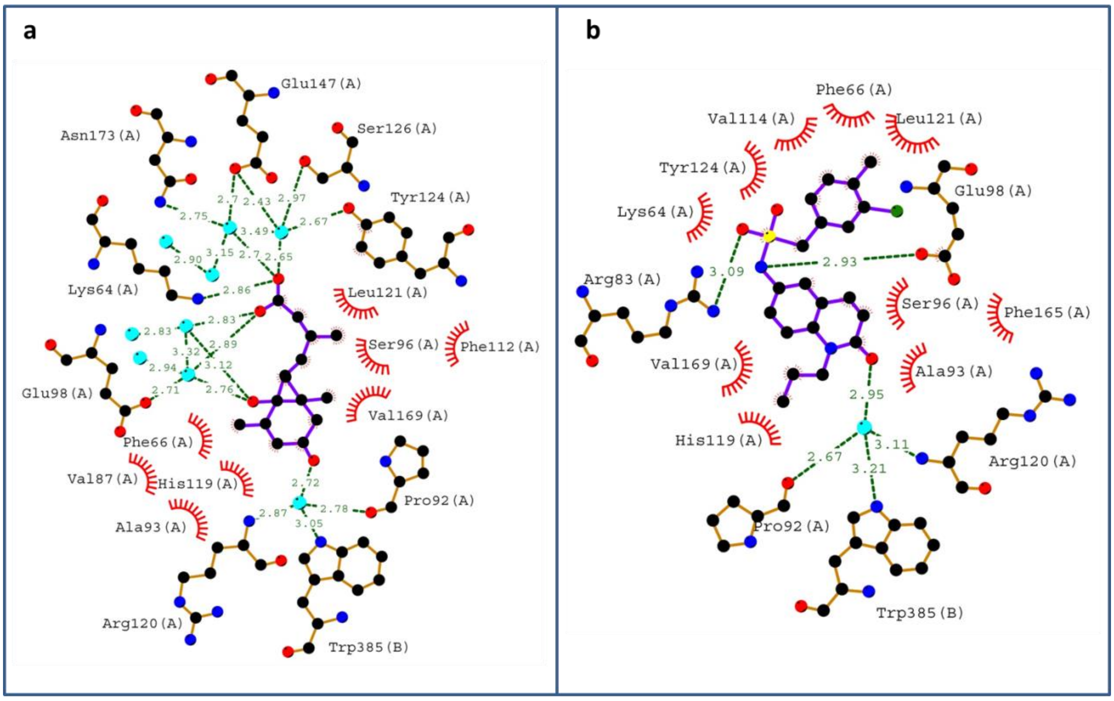

PYL2-ABA-HAB1

PYL2-AMC1β-HAB1

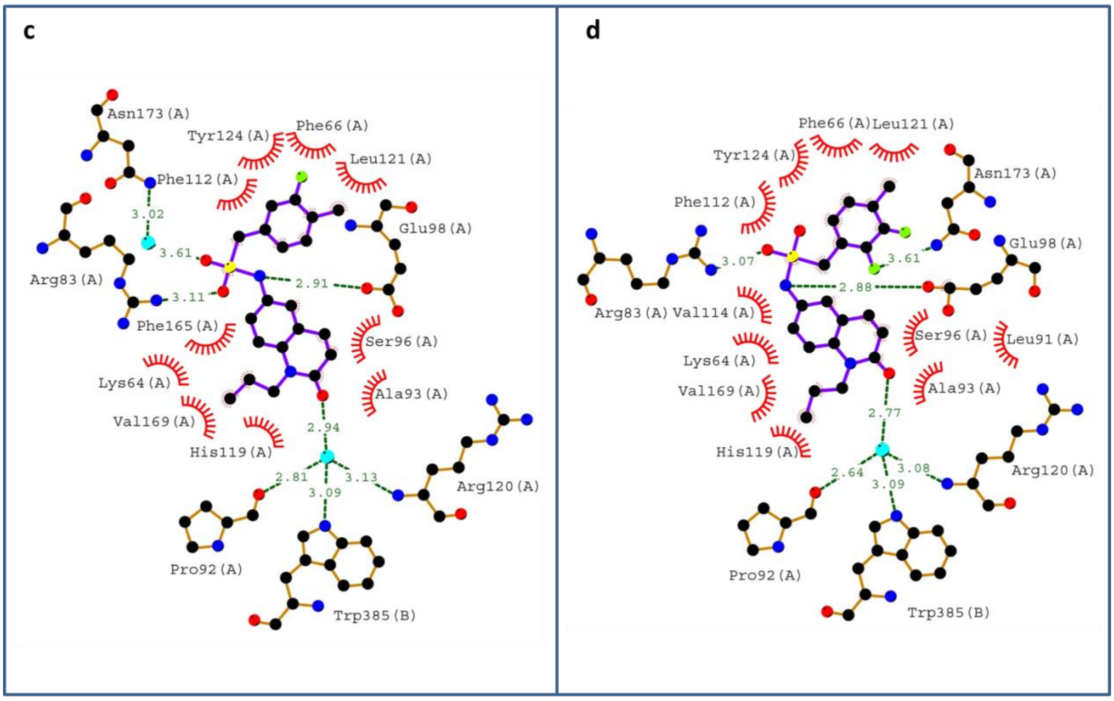

PYL2-AMF1β-HAB1

PYL2-AMF2α-HAB1

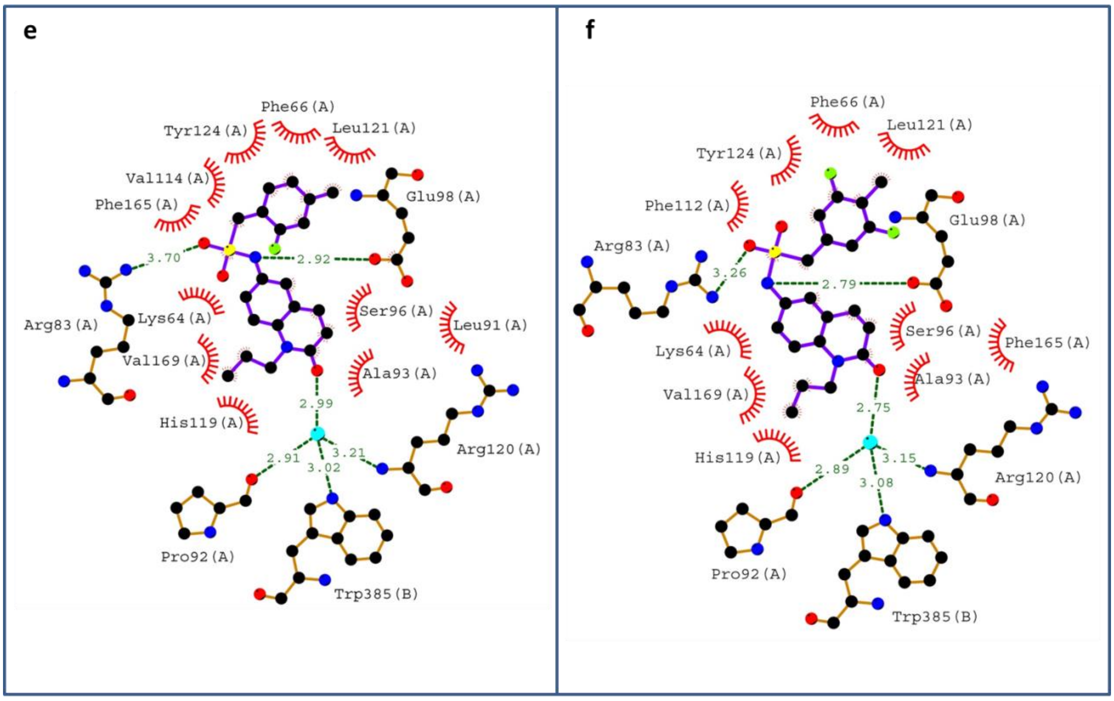

PYL2-AMF1α-HAB1 PYL2-AMF2β-HAB1

Supplementary Figure 2. Two-dimensional structural schematic of interactions between PYL2 binding-pocket residues and (+)-ABA (a), AMC1β (b), or AMFs (c-f).

The schematics show the hydrogen bonds (dashed lines) between residues in the PYL2 binding pocket (A) or in HAB1 (B) and ABA (a), AMC1β (b), or AMFs (c-f). Green-, red-, yellow-, blue-, and turquoise-filled circles represent fluorine/chloride atoms, oxygen atoms, sulfur atoms, nitrogen atoms, and water molecules, respectively. The number represents the distance (Å) between two atoms/molecules.

Supplementary Figure 3

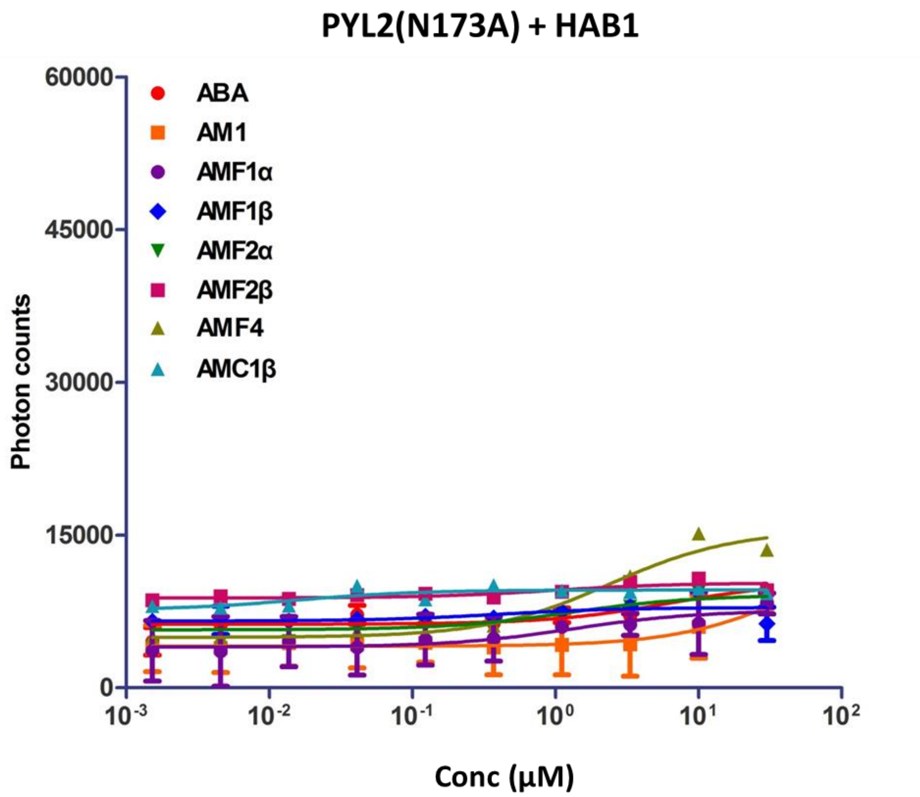

Supplementary Figure 3. Chemical-PYL2 interactions are nearly abolished by the N173A mutation.

Agonist dose-response curves show that interactions between HAB1 and mutated PYL2 (N173A) in the presence of all five AMFs, AMC1β and AM1 are nearly abolished as in the presence of (+)-ABA.

Supplementary Figure 4

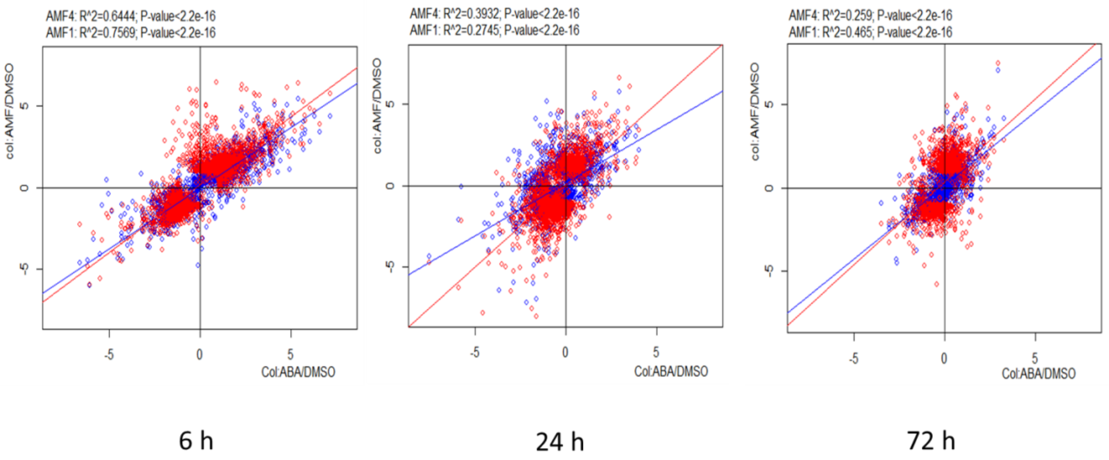

Supplementary Figure 4. AMFs induce ABA-responsive gene expression in wild type *Arabidpopsis*.

Transcript-level responses induced by AMF1β and AMF4 are highly correlated with those induced by ABA. Scatterplot log2-transformed values of differentially expressed genes (DEGs) responsive to AMF treatment (Y axis) and ABA treatment (X axis) relative to the DMSO control. Blue dots represent the DEGs responsive to AMF1β, while red dots represent the DEGs responsive to AMF4. The Spearman correlation coefficient (r) and P-value are shown.

Supplementary Figure 5

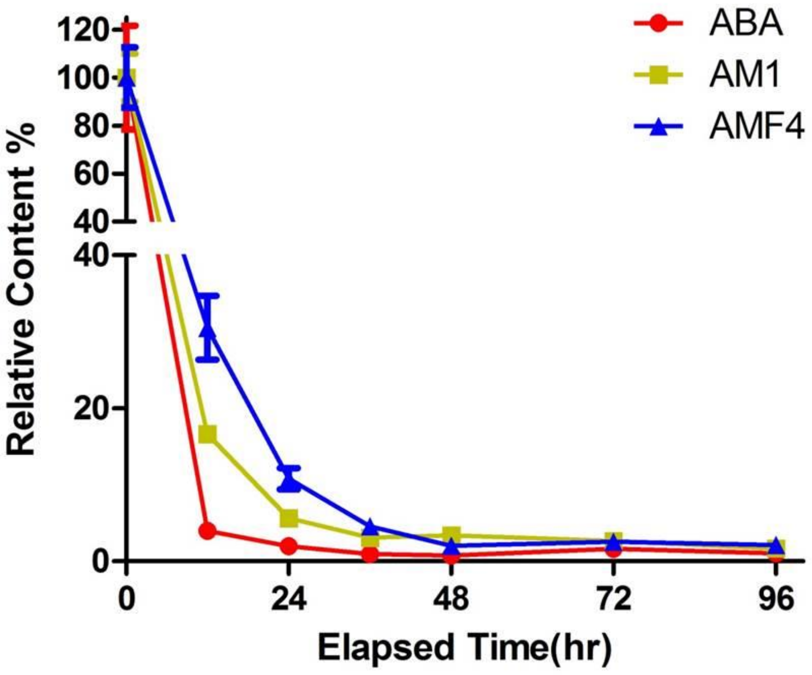

Supplementary Figure 5. *In-vivo* stability of ABA, AM1 and AMF4.

Compound contents in plants were normalized to the initial contents after treatments. Values are the mean contents and error bars indicate the standard deviation of three biological replicates per treatment.

Supplementary Figure 6

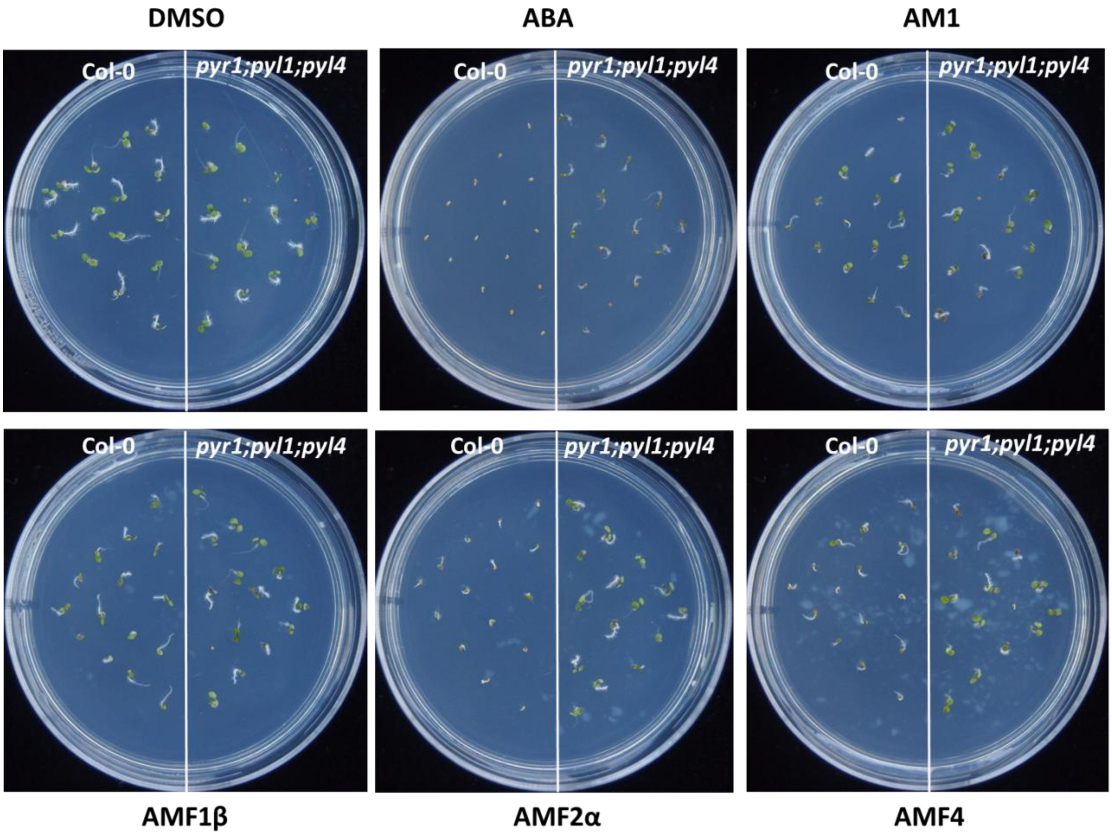

Supplementary Figure 6. AMFs inhibit of seed germination.

Seeds of the wild type (Col-0, left half of each plate) and the PYL triple mutant (*pyr1;pyl1;pyl4*, right half of each plate) are grown on the same half-strength MS (1% sucrose) plate containing the indicated compounds. The concentration is 1 µM for all AMFs and (+)-ABA, and (+)-ABA and DMSO are used as the positive and negative controls, respectively. Plates are photographed 4 days after the seeds of the triple mutant begin to germinate.

Supplementary Figure 7

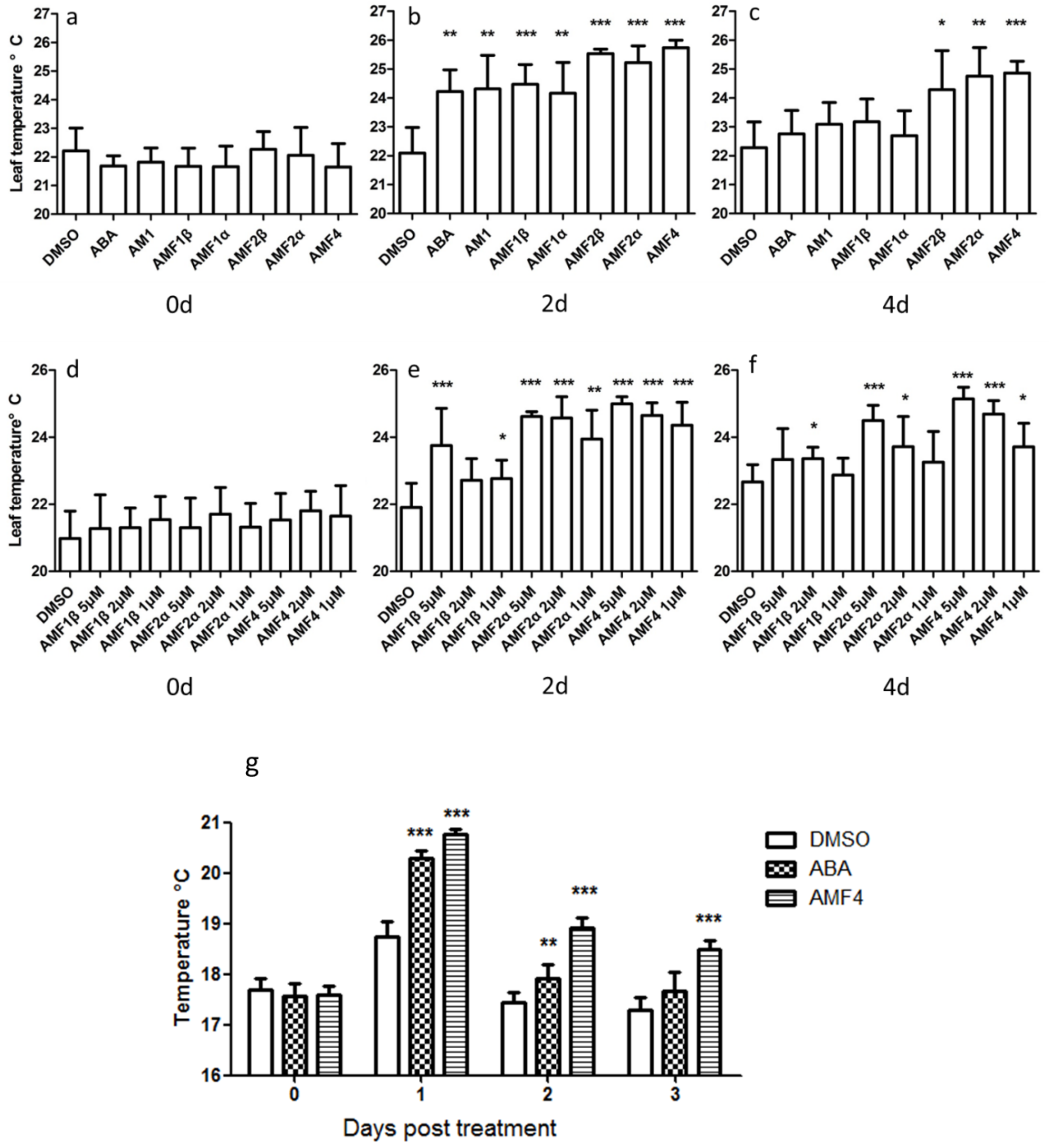

Supplementary Figure 7. Quantification of leaf temperature.

Leaf surface temperatures (older leaves for soybean) in Figure 5 were quantified from six independent areas of each plant in each figure, with a-c, d-f and g corresponding to a, b and c in Figure 5, respectively. Error bars indicate the standard deviation of six independent areas. The asterisks indicate statistical significance at **P* < 0.05, ***P* < 0.01 and ****P* < 0.01.

Supplementary Figure 8

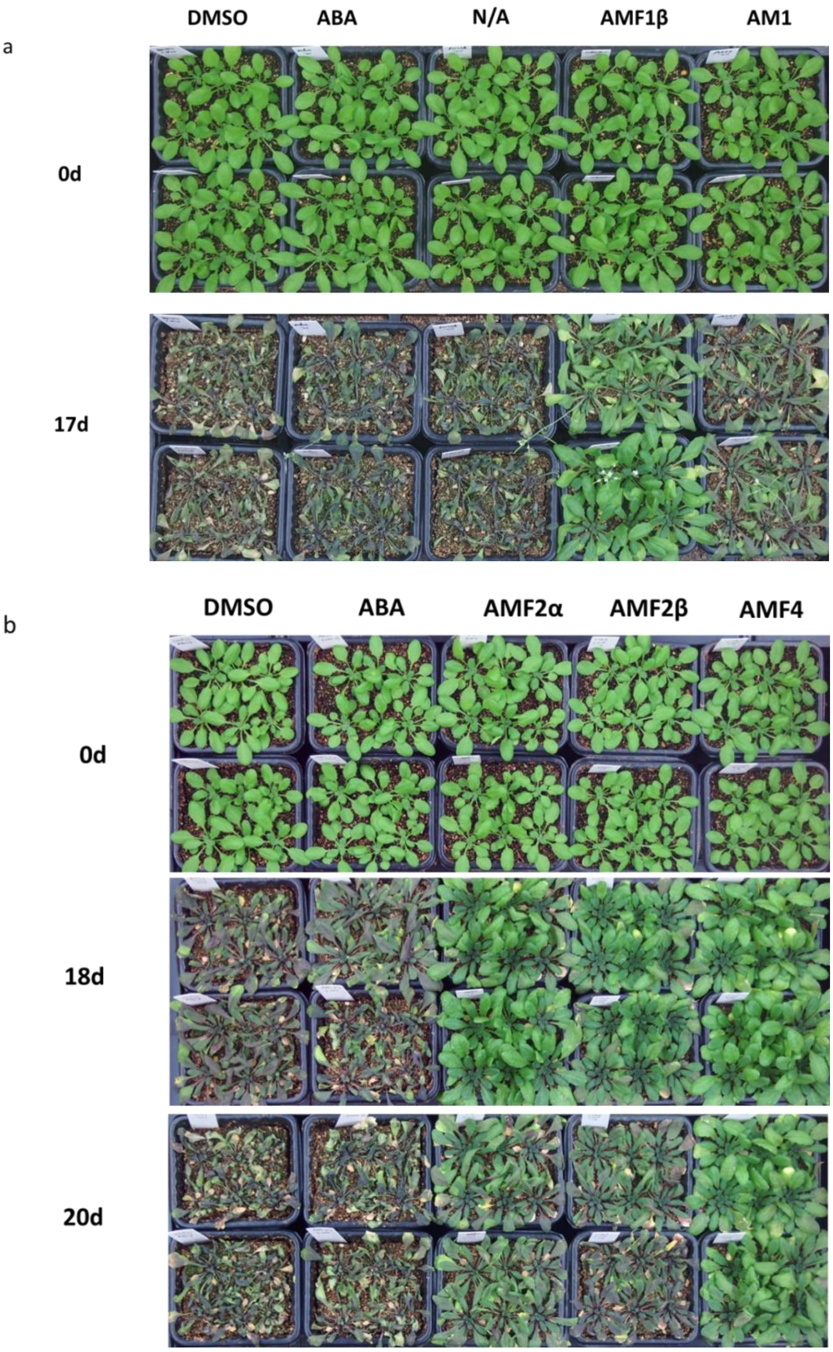

Supplementary Figure 8. AMFs increase drought resistance of *Arabidopsis* Col-0 plants.

(a) Two-week-old *Arabidopsis* wild-type plants are subjected to drought (watering was stopped) and are then treated with 10 μM solutions of (+)-ABA, AM1, or

AMF1β once per week for 2 weeks. DMSO is used as the control. The plants are photographed before watering is stopped (top panel) and at 17 days after watering is stopped (bottom panel). N/A represents plants from an unrelated experiment.

(b) Two-week-old *Arabidopsis* wild-type plants are subjected to drought (watering was stopped) and are then treated with 5 μM solutions of (+)-ABA, AMF2α, AMF2β, or AMF4 once per week for 2 weeks. DMSO is used as the control. The plants are photographed before watering is stopped (top panel) and at 18 days (middle panel) and 20 days (bottom panel) after watering is stopped.

Supplementary Figure 9

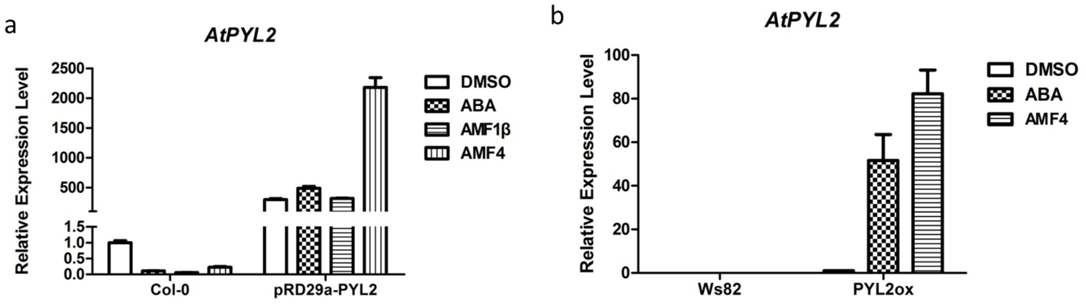

Supplementary Figure 9. Analysis of gene expression in *Arabidopsis* and soybean p*RD29a-PYL2* transgenic plants in response to treatment with ABA or AMFs.

Arabidopsis and soybean p*RD29a-PYL2* transgenic plants (pRD29a-PYL2 for *Arabidopsis* and PYL2ox for soybean) are analyzed by quantitative real-time PCR. Expression levels of *AtPYL2* are measured in *Arabidopsis* (a) or soybean (b) transgenic lines before and 6 h post-treatment with DMSO, ABA, or AMFs. *AtACT7* and *GmACT2* are used as internal controls in *Arabidopsis* and soybean, respectively.

Supplementary Figure 10

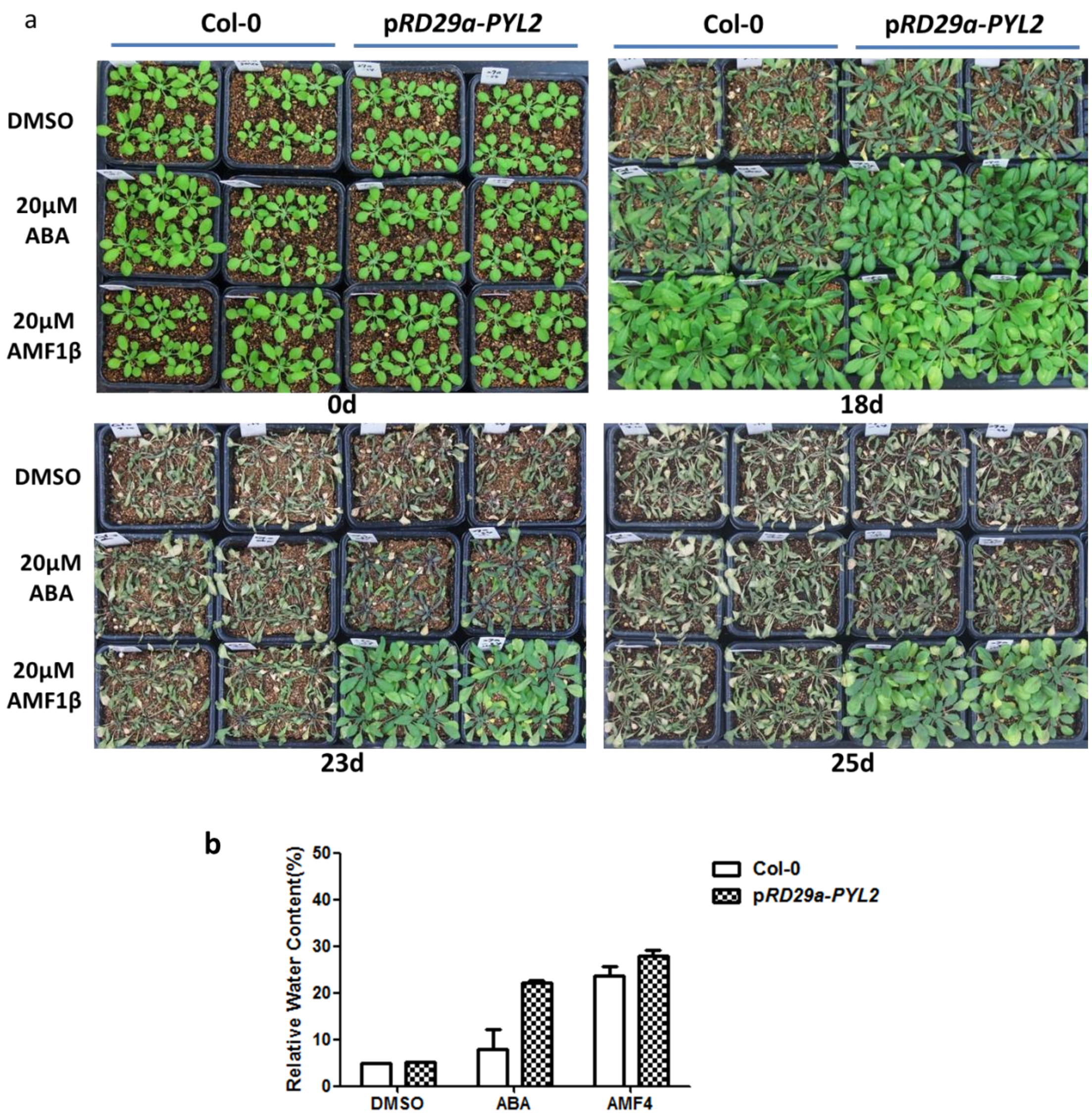

Supplementary Figure 10. AMFs dramatically increase drought resistance in *PYL2* overexpression *Arabidopsis* plants.

(a) *Arabidopsis* wild-type plants (Col-0) and *PYL2* transformants driven by *RD29a* promoter (p*RD29a-PYL2*) are grown under short-day conditions for 2 weeks before watering is stopped. Plants are subsequently treated with DMSO (control), 20 μM (+)-ABA, or 20 μM AMF1β once per week for another 2 weeks. Plants are photographed before watering is stopped (top left panel), 18 days after watering

is stopped (top right panel), 23 days after watering is stopped (bottom left panel, 1 day before watering is resumed), and 1 day after watering is resumed (bottom right panel).

(b) Relative soil water content of plants in Figure 7a is determined as mass of water in soil/mass of oven-dry soil *100%. Values are means of 6 pots for each combination of plant line and treatment at 18 days after watering is stopped. Error bars indicate SD.

Supplementary Table 1. Primer Sequences

Primer	Sequence (5'-3')
AtPYL2 qF	CACGGTGGTTCTTGAATCTTAC
AtPYL2 qR	CATCATCATGCATAGGTGCAGA
AtACT7 qF	CATTCAATGTCCCTGCCATGT
AtACT7 qR	GGTTGTACGACCACTGGCATAG
GmACT2 qF	CTTCCCTCAGCA CCTTCCAA
GmACT2 qR	GGTCCAGCTTTCA CACTCCAT
ASNTA-FP	CAA ACT CGC CCT TCA GAA ACT CGG AGT TGC CGC CAC
ASNTA-RP	CTG AAG GGC GAG TTT GAC GAC AGT GTC CAC AAA C

Supplementary Table 2. Statistics of data sets and structure refinement

Crystal ID	PYL2-AMF1β-HAB1	PYL2-AMF2α-HAB1	PYL2-AMF1α-HAB1	PYL2-AMF2β-HAB1	PYL2-AMF4-HAB1	PYL2-AMC1β-HAB1
PDB code	5VRO	5VS5	5VR7	5VSQ	5VSR	5VT7
**Data statistics**						
Beam line	SSRF-BL17U1	SSRF-BL17U1	SSRF-BL17U1	SSRF-BL17U1	SSRF-BL17U1	SSRF-BL17U1
Space group	$P\ 2_1\ 2_1\ 2_1$	$P\ 2_1\ 2_1\ 2_1$	$P\ 2_1\ 2_1\ 2_1$	$P\ 2_1\ 2_1\ 2_1$	$P\ 2_1\ 2_1\ 2_1$	$P\ 2_1\ 2_1\ 2_1$
Resolution range, Å	50—2.26 (2.30-2.26)*	72.9—2.33 (2.46-2.33)	50—2.62 (2.67-2.62)	72.9—2.62 (2.76-2.62)	72.4—2.62 (2.76-2.62)	50.0-2.62 (2.67-2.62)
Cell parameters a, b, c, Å α, β, γ, °	61.3,67.9,145.9 90, 90, 90	62.3,68.6,145.7 90, 90, 90	61.2,67.3,146.3 90, 90, 90	61.1,67.4,145.7 90, 90, 90	61.2,64.4,144.8 90, 90, 90	61.1,66.5,145.4 90, 90, 90
Total/ Unique reflections	392913/29256	130353/24782	249832/18972	187385/18843	110346/17605	250857/18330
Completeness, %	99.3 (96.8)	99.6 (99.3)	99.9 (97.9)	100 (100)	99.9 (100)	99.6 (98.0)
Mean I/σ	73.1 (9.7)	9.1 (1.5)	32.7 (7.4)	41.2 (10.3)	10.3 (1.7)	29.2 (7.4)
Multiplicity	13.1 (12.7)	5.5 (5.3)	13.2 (12.3)	9.9 (11.4)	5.7 (6.0)	13.7 (13.9)
Rmerge	0.076 (0.262)	0.151 (1.783)	0.103 (0.474)	0.108 (0.436)	0.176 (2.221)	0.113 (0.517)
CC1/2	0.998 (0.988)	0.998 (0.988)	0.998 (0.988)	0.997 (0.977)	0.993 (0.559)	0.998 (0.970)
**Refinement**						
Resolution, Å	39.5—2.26	72.9—2.33	33.7—2.61	39.4—2.62	38.6—2.62	38.3—2.62
No. reflections	29199	26860	18904	18775	17387	18276
No. residues	482	473	487	412	485	485
No.solvent molecules	164	247	45	79	18	66
No. of non-H atoms	3770	3728	3876	3825	3818	3864
Rcryst	21.5%	21.5%	21.6%	20.4%	23.9%	18.9%
Rfree	24.3%	24.4%	24.5%	24.5%	27.0%	22.6%
Rmsd** bonds, Å	0.004	0.005	0.004	0.004	0.005	0.008
Rmsd** angles, °	0.992	0.887	0.984	0.976	1.021	1.169
Average B factor, $Å^2$	40.7	35.5	42.7	37.6	40.6	36.3
Ramachandran, % Favored/Outliers	98.1/0.0	97.8/0.0	98.5/0.0	98.3/0.0	97.7/0.0	98.7/0.0

*Values in parentheses are for highest-resolution shell.

**Rmsd is the root-mean-square deviation from ideal geometry of protein.

Supplementary Note 1. Chemical Synthesis

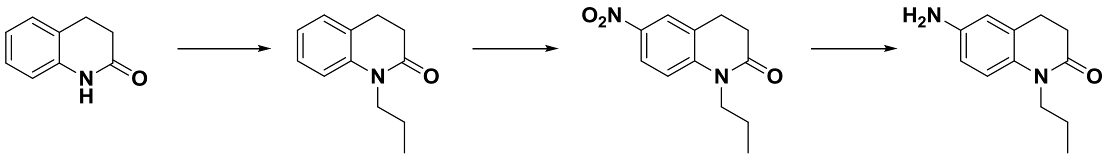

a1 a2 a3 a4

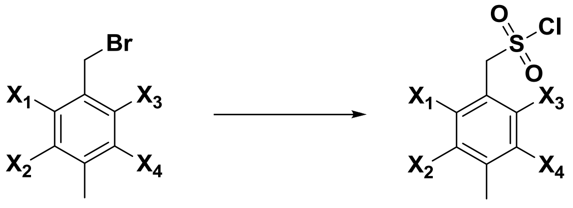

b1-6 b7-12

b1/b7: X1 = X2 = X3 = X4 = F
b2/b8: X1 = X2 = H; X3 = X4 = F
b3/b9: X1 = X2 = X3 = H; X4 = F
b4/b10: X1 = X3 = H; X2 = X4 = F
b5/b11: X1 = F; X2 = X3 = X4 = H
b6/b12: X1 = X2 = X3 = H; X4 = Cl

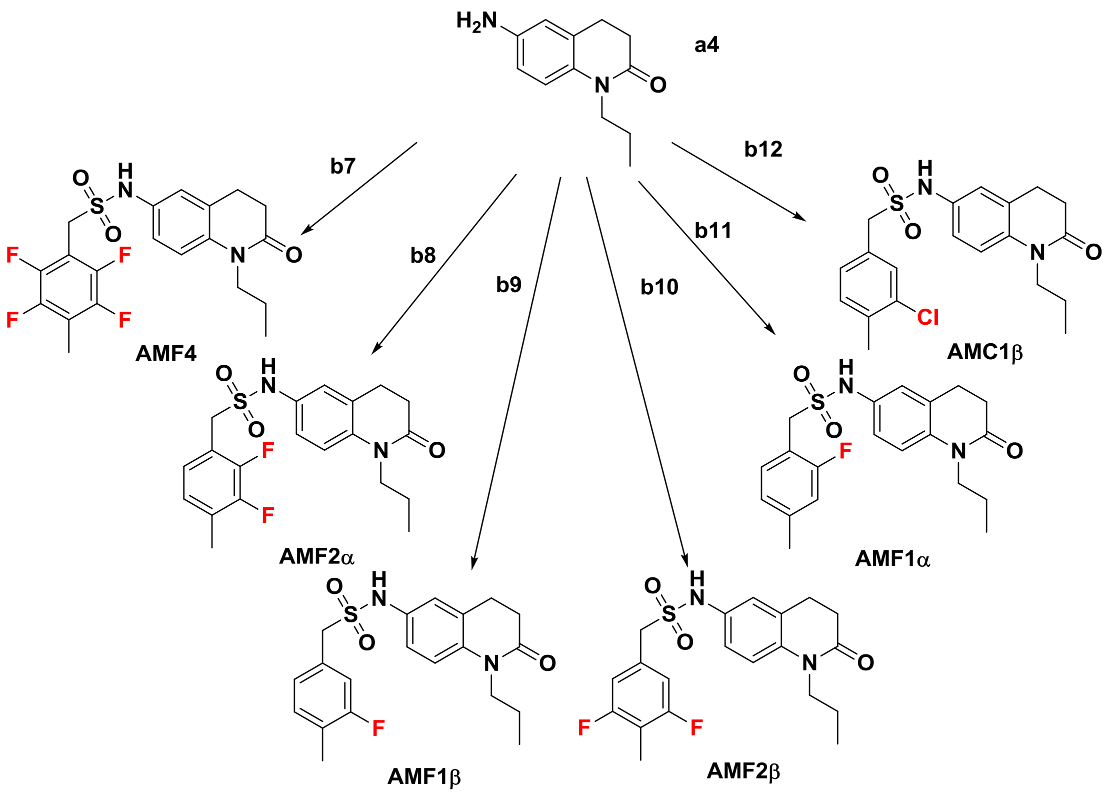

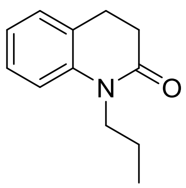

1-propyl-3,4-dihydroquinolin-2(1H)-one (a2)

3,4-dihydroquinolin-2(1*H*)-one (a1, 4.0 g , 27.2 mmol) and anhydrous DMF (60 ml) were added into a three-neck flask and stirred in an ice-water bath. Sodium hydride (32.7 mmol, 1.2 equiv) was added gradually at such a rate that the inner temperature was maintained less than 5 °C. The mixture was stirred for another 0.5 h. The solution of 1-iodopropane (32.7 mmol, 1.2 equiv) and anhydrous DMF (20 ml) was added dropwise. After the addition, the cooling bath was removed. The reaction was maintained for 8 h at room temperature and then quenched by adding 100 ml of ammonium chloride (aq.), extracted with ethyl acetate for three times. The organic phase was dried over anhydrous Na_2SO_4 and concentrated under reduced pressure. The crude product was purified by chromatographic column on silca gel, giving 1-propyl-3,4-dihydroquinolin-2(1H)-one (a2) as a brown liquid, 4.4 g (86%). ^{1}H NMR (400 MHz, $CDCl_3$): δ 7.24-6.98 (m, 4H), 3.89 (m, 2H), 2.89 (m, 2H), 2.64 (m, 2H), 1.66 (m, 2H), 0.97 (t, *J* = 7.6 Hz, 3H) ppm.

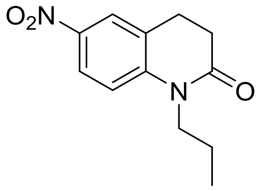

6-nitro-1-propyl-3,4-dihydroquinolin-2(1H)-one (a3)

The 1-propyl-3,4-dihydroquinolin-2(1H)-one (a2, 4.0 g, 21.2 mmol) were dissolved in DCM (100 ml). The solution was stirred and cooled in an ice-water bath. Then fuming HNO_3 (1.5 equiv) was added to the solution dropwise. The mixture was stirred for 2 h. Then the reaction mixture was poured into ice water. The suspension was separated by vacuum filtration. The crude product was purified with recrystallization from ethanol, giving 6-nitro-1-propyl-3,4-dihydroquinolin-2(1H)-one (a3) as a light yellow solid, 3.6 g (72%); ^{1}H NMR (400 MHz, $CDCl_3$): δ 8.14(m, 1H), 8.07(m, 1H), 7.06 (d, *J* = 8.8 Hz, 1H), 3.93 (m, 2H), 3.00 (m, 2H), 2.71 (m, 2H), 1.68 (m, 2H), 0.98 (t, *J* = 7.6 Hz, 3H) ppm.

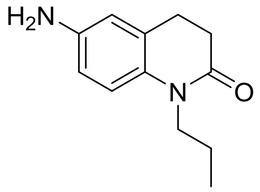

6-amino-1-propyl-3,4-dihydroquinolin-2(1*H*)-one (a4)

A flask was charged with 6-nitro-1-propyl-3,4-dihydroquinolin-2(1H)-one (a3, 4.0 g, 17.1

mmol) , methanol (150 ml), and Pd/C (wet, 10%, 20 mg) under a nitrogen atmosphere. Then the flask was evacuated and recharged with hydrogen three times. The mixture was allowed to stir for 8 h at room temperature. The reaction solution was filtered under vacuum and washed with methanol. The organic filtrate was collected and evaporated under vacuum to give 6-amino-1-propyl-3,4-dihydroquinolin-2(1*H*)-one (a4) as a brown solid, 3.1 g (88%).

^{1}H NMR (400 MHz, $CDCl_3$): δ 6.79 (d, *J* = 8.8 Hz, 1H), 6.56 (dd, *J* = 8.4, 2.8 Hz, 1H), 6.52 (d, *J* = 2.8 Hz, 1H), 3.83 (m, 2H), 3.48 (br, 2H), 2.78 (m, 2H), 2.58 (m, 2H), 1.63 (m, 2H), 0.93 (t, *J* = 7.2 Hz) ppm.

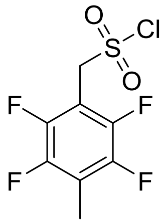

(2,3,5,6-tetrafluoro-4-methylphenyl)methanesulfonyl chloride (b7)

This is a general procedure for synthesis of b7-12.

A flask was charged with 1-(bromomethyl)-2,3,5,6-tetrafluoro-4-methylbenzene (b1, 2.0 g, 7.8 mmol), thiourea (7.8 mmol), and ethanol (80 ml). The mixture was allowed to stir under reflux for 3 h. After completion, the reaction solution was concentrated to obtain a white solid. 20 ml of MeCN and 20 ml of conc. HCl were sequentially added into the flask. The mixture was kept stirring until all solid dissolved, then $NaClO_2$ (5.0 equiv) was added gradually at such a rate that the inner temperature was maintained less than 20 °C. After the addition, the mixture was stirred for another 3 h, then the reaction was stopped by adding 100 ml of cold water. The resulting mixture was extracted with ethyl acetate for three times. The organic phase was dried over $MgSO_4$, and then concentrated in vacuum, giving (2,3,5,6-tetrafluoro-4-methylphenyl)methanesulfonyl chloride (b7) as a light yellow solid.

^{1}H NMR (400 MHz, DMSO-*d*6): δ 3.94 (s, 2H), 2.28 (s, 3H) ppm.

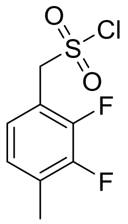

(2,3-difluoro-4-methylphenyl)methanesulfonyl chloride (b8)

^{1}H NMR (400 MHz, DMSO-*d*6): δ 7.04 (m, 2H), 3.81 (s, 2H), 2.23 (s, 3H) ppm

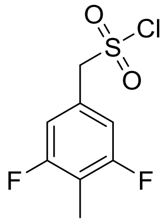

(3,5-difluoro-4-methylphenyl)methanesulfonyl chloride (b10)

^{1}H NMR (400 MHz, DMSO-*d*6): δ 6.97(m, 2H), 3.77 (s, 2H), 2.11 (s, 1H) ppm.

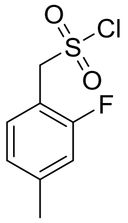

(2-fluoro-4-methylphenyl)methanesulfonyl chloride (b11)

^{1}H NMR (400 MHz, DMSO-*d*6): δ 7.62-7.15 (m, 3H), 3.77(s, 2H), 2.28 (s, 3H) ppm.

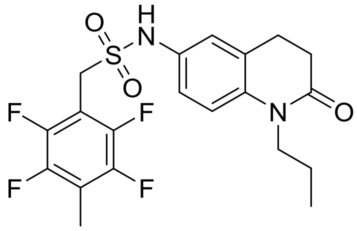

AMF4

6-amino-1-propyl-3,4-dihydroquinolin-2(1*H*)-one (a4, 1.0 g, 4.9 mmol), DMF (20ml), K_2CO_3 (3 equiv), and (2,3,5,6-tetrafluoro-4-methylphenyl)methanesulfonyl chloride (b7, 5.9 mmol, 1.2 equiv) were sequentially added to a 50 ml flask. The mixture was allowed to stir under a nitrogen atmosphere for 12 h at room temperature. The reaction was quenched by adding cold water and then extracted with ethyl acetate for three times. The organic phase was dried over Na_2SO_4, concentrated in vacuum and purified by chromatographic column on silca gel, giving AMF4 as a light yellow solid.

^{1}H NMR (400 MHz, DMSO-*d*6): δ 10.07 (s, 1H), 7.08-7.05 (m, 3H), 4.58 (s, 2H), 3.81 (t, *J* = 7.2 Hz, 2 H), 2.80 (t, *J* = 6.8 Hz, 2H), 2.52 (t, in DMSO peak, 2H), 2.25 (s, 3H), 1.52 (m, 2H)，0.88 (t, *J* = 7.6 Hz, 3H) ppm; ^{13}C NMR (101MHz, $CDCl_3$) δ 170.1, 146.1 (m), 143.7 (m), 137.0, 131.4, 127.9, 120.4, 119.5, 117.8 (t, *J* = 19.0 Hz), 115.7, 105.6 (t, *J* = 17.1 Hz), 45.9, 43.8, 31.6, 25.5, 20.4, 11.2, 7.7 ppm. HRMS (m/z): $[M]^+$ calcd. for $C_{20}H_{20}F_4N_2O_3S$, 444.1131; found, 444.1138.

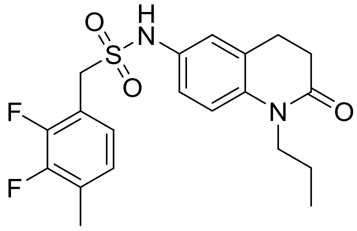

AMF2α

6-amino-1-propyl-3,4-dihydroquinolin-2(1*H*)-one (a4, 1.0 g, 4.9 mmol), DMF (20ml), K_2CO_3 (3 equiv), and (2,3-difluoro-4-methylphenyl)methanesulfonyl chloride (b8, 5.9 mmol, 1.2 equiv) were sequentially added to a 50 ml flask. The mixture was allowed to stir under a nitrogen atmosphere for 4 h at room temperature. The reaction was quenched by adding cold water and

then extracted with ethyl acetate for three times. The organic phase was dried over Na_2SO_4, concentrated in vacuum and purified by chromatographic column on silca gel, giving AMF2α as a light yellow solid.

^1H NMR (400 MHz, $CDCl_3$): δ 7.09-6.92 (m, 5H), 6.79 (s, 1H), 4.42 (s, 2H), 3.89 (t, *J* = 7.6 Hz, 2H), 2.87 (t, *J* = 6.8 Hz, 2H), 2.65 (m, 2H), 2.29 (s, 3H), 1.68 (m, 2H)， 0.98 (t, *J* = 7.6 Hz, 3H) ppm; ^{13}C NMR (101 MHz, $CDCl_3$): δ 169.9, 137.2, 131.2, 128.5, 128.4, 127.2, 126.2 (br), 125.9 (br), 120.9, 120.0, 115.6, 115.5, 115.4, 50.8, 43.7, 31.6, 25.5, 20.4, 14.3, 11.2 ppm. HRMS (m/z): $[M]^+$ calcd. for $C_{20}H_{22}F_2N_2O_3S$, 408.1319; found, 408.1324.

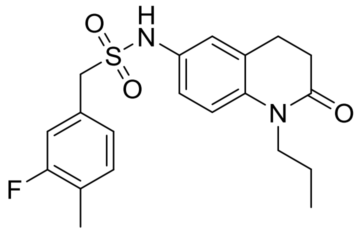

AMF1β

6-amino-1-propyl-3,4-dihydroquinolin-2(1*H*)-one (a4, 1.0 g, 4.9 mmol), DMF (20ml), K_2CO_3 (3 equiv) and (3-fluoro-4-methylphenyl)methanesulfonyl chloride (b9, 5.9 mmol, 1.2 equiv) were sequentially added to a 50 ml flask. The mixture was allowed to stir under a nitrogen atmosphere for 4 h at room temperature. The reaction was quenched by adding cold water and then extracted with ethyl acetate for three times. The organic phase was dried over Na_2SO_4, concentrated in vacuum and purified by chromatographic column on silca gel, giving AMF1β as a light yellow solid.

^1H NMR (400 MHz, $CDCl_3$): δ 7.18 (t, *J* = 8.4 Hz, 1H), 7.06-6.94 (m, 4H), 6.75 (s, 1H), 4.29 (s, 2H),3.89 (m, 2H), 2.88 (m, 2H), 2.65 (m, 2H), 2.27 (d, *J* = 1.2 Hz, 3H), 1.67 (m, 2H), 0.99 (t, *J* = 6.4 Hz. 3H); ^{13}C NMR (101 MHz, $CDCl_3$): δ = 169.8, 161.5 (d, *J* = 245 Hz), 137.1, 131.7 (d. *J* = 5.4 Hz), 131.3, 128.1, 127.7 (*J* = 8.0 Hz), 126.3 (d, *J* = 3.3 Hz), 125.9 (d, *J* = 16.9 Hz), 120.9, 120.0, 117.3 (d, *J* = 23.2 Hz), 115.7, 56.9, 43.7, 31.6, 25.6, 20.4, 14.4, 11.2 ppm. HRMS (m/z): $[M]^+$ calcd. for $C_{20}H_{23}FN_2O_3S$, 390.1413; found, 390.1419.

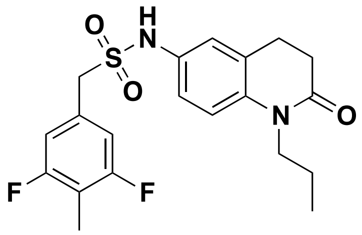

AMF2β

6-amino-1-propyl-3,4-dihydroquinolin-2(1*H*)-one (a4, 1.0 g, 4.9 mmol), DMF (20ml), K_2CO_3 (3 equiv) and (3,5-difluoro-4-methylphenyl)methanesulfonyl chloride (b10, 5.9 mmol, 1.2 equiv) were sequentially added to a 50 ml flask. The mixture was allowed to stir under a nitrogen atmosphere for 4 h at room temperature. The reaction was quenched by adding cold water and then extracted with ethyl acetate for three times. The organic phase was dried over Na_2SO_4, concentrated in vacuum and purified by chromatographic column on silca gel, giving AMF2β as a

light yellow solid.

^{1}H NMR (400 MHz, DMSO-*d*6): δ 9.75(s, 1H), 7.09-6.96 (m, 5H), 4.49 (s, 2H), 3.81(t, *J* = 7.2 Hz, 2H), 2.80 (t, *J* = 6.8 Hz, 2H), 2.53 (t, in DMSO peak, 2H), 2.13 (s, 3H), 1.54 (m, 2H)，0.88 (t, *J* = 7.2 Hz, 3H) ppm; ^{13}C NMR (101 MHz, DMSO-*d*6): δ 169.3, 161.0 (d, *J* = 242 Hz), 136.0, 132.9, 130.3 (t, *J* = 10.4 Hz), 127.7, 120.2, 119.5, 116.1, 114.1 (d, *J* = 27 Hz), 113.0 (t, *J* = 21.3 Hz), 56.6, 42.9, 31.7, 25.4, 20.4, 11.5, 7.2 ppm. HRMS (m/z): $[M]^+$ calcd. for $C_{20}H_{22}F_2N_2O_3S$, 408.1319; found, 408.1315.

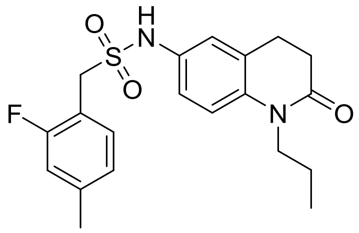

AMF1α

6-amino-1-propyl-3,4-dihydroquinolin-2(1*H*)-one (a4, 1.0 g, 4.9 mmol), DMF (20 ml), K_2CO_3 (3 equiv) and (2-fluoro-4-methylphenyl)methanesulfonyl chloride (b11, 5.9 mmol, 1.2 equiv) were sequentially added to a 50 ml flask. The mixture was allowed to stir under a nitrogen atmosphere for 16 h at room temperature. The reaction was quenched by adding cold water and then extracted with ethyl acetate for three times. The organic phase was dried over Na_2SO_4, concentrated in vacuum and purified by chromatographic column on silca gel, giving AMF1α as a light yellow solid.

^{1}H NMR (400 MHz, $CDCl_3$): δ 7.33 (d, *J* = 6.8 Hz, 1H), 7.09-6.92 (m, 5H), 4.35 (s, 2H), 3.88 (m, 2H), 2.86 (t, *J* = 6.4 Hz, 2H), 2.64 (m, 2H), 2.33 (s, 3H), 1.66 (m, 2H), 0.97 (t, *J* = 7.2 Hz, 3H) ppm; ^{13}C NMR (101 MHz, $CDCl_3$): δ 169.9, 159.4 (d, *J* = 247 Hz), 139.4 (d, *J* = 8 Hz), 137.0，132.3 (d, *J* = 3.1), 131,2, 129.6, 127,9, 120.8, 119.9, 118.0 (d, *J* = 23.1 Hz), 115.8, 115.0 (d, *J* = 19.3Mz), 50.5, 43.7, 31.6, 25.6, 20.4, 20.2, 11.2 ppm. HRMS (m/z): $[M]^+$ calcd. for $C_{20}H_{23}FN_2O_3S$, 390.1413; found, 390.1424.

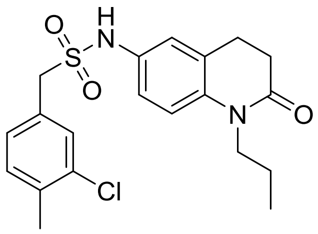

AMC1β

6-amino-1-propyl-3,4-dihydroquinolin-2(1*H*)-one (a4, 1.0 g, 4.9 mmol), DMF (20 ml), K_2CO_3 (3 equiv) and (3-chloro-4-methylphenyl)methanesulfonyl chloride (b12, 5.9 mmol, 1.2 equiv) were sequentially added to a 50 ml flask. The mixture was allowed to stir under a nitrogen atmosphere for 16 h at room temperature. The reaction was quenched by adding cold water and then extracted with ethyl acetate for three times. The organic phase was dried over Na_2SO_4, concentrated in vacuum and purified by chromatographic column on silca gel, giving AMC1β as a light yellow solid. b6 as the starting material of b12 contains 2-Cl isomer about 25-35%.

^{1}H NMR (400 MHz, $CDCl_3$): δ 7.39-7.32 (m, 1H), 7.25-6.88 (m, 5H), 4.28 (m, 2H), 3.89 (m, 2H), 2.86

(m, 2H), 2.66 (m, 2H), 2.32 (s, 3H), 1.67 (m, 2H), 0.99 (t, *J* = 7.2 Hz, 3H) ppm; ^{13}C NMR (101 MHz, $CDCl_3$): δ 169.9, 137.0, 136.7, 135.3, 134.6, 133.4, 131.3, 129.5, 128.1, 126.9, 120.7, 119.8, 115.7, 57.0, 43.8, 31.6, 25.6, 20.7, 20.0, 11.3 ppm. HRMS (m/z): $[M]^+$ calcd. for $C_{20}H_{23}ClN_2O_3S$, 406.1119; found, 406.1128.
